# C1GALT1 expression predicts poor survival in osteosarcoma and is crucial for ABCC1 transporter‐mediated doxorubicin resistance

**DOI:** 10.1002/path.6384

**Published:** 2025-01-22

**Authors:** Chun‐Wei Liu, Jing‐Hui Huang, Hsiu‐Hao Chang, Chia‐Hua Chen, Yi‐Huan Tsai, Wei‐Li Chen, Jung‐An Lin, Hsiu‐Ling Chang, Cheng‐Chang Chen, Mei‐Chun Lin, Min‐Chuan Huang, Neng‐Yu Lin

**Affiliations:** ^1^ Graduate Institute of Anatomy and Cell Biology National Taiwan University College of Medicine Taipei Taiwan; ^2^ Department of Biochemistry and Molecular Medicine National Dong Hwa University Hualien Taiwan; ^3^ Department of Pediatrics National Taiwan University Hospital, National Taiwan University College of Medicine Taipei Taiwan; ^4^ Department of Anatomy, School of Medicine Chang Gung University Taoyuan Taiwan; ^5^ Neuroscience Research Center Chang Gung Memorial Hospital, Linkou Medical Center Taoyuan Taiwan; ^6^ Graduate Institute of Biomedical Sciences, College of Medicine Chang Gung University Taoyuan Taiwan; ^7^ Department of Laboratory Medicine National Taiwan University Hospital Taipei Taiwan; ^8^ Department of Clinical Laboratory Sciences and Medical Biotechnology, College of Medicine National Taiwan University Taipei Taiwan; ^9^ National Taiwan University Cancer Center Taipei Taiwan

**Keywords:** C1GALT1, ABCC1, doxorubicin, osteosarcoma, chemoresistance, O‐glycosylation

## Abstract

Osteosarcoma is an aggressive bone malignancy with a high propensity for drug resistance and metastasis, leading to poor clinical outcomes. This study investigates the role of core 1 β1,3‐galactosyltransferase 1 (C1GALT1) in osteosarcoma, focusing on its implications in chemoresistance. Our findings reveal that high expression of C1GALT1 is associated with advanced stages, adverse overall survival, and increased recurrence rates. Elevated levels of C1GALT1 were observed in doxorubicin‐selected osteosarcoma cells, where its suppression significantly promoted doxorubicin‐induced apoptosis and reduced drug efflux. Pharmacological inhibition of C1GALT1 using itraconazole replicated these effects, suggesting a potential therapeutic strategy to overcome chemoresistance. Additionally, we identified the involvement of the ATP‐binding cassette (ABC) transporter ABCC1 in the drug‐resistance phenotype mediated by C1GALT1. C1GALT1‐mediated O‐glycan changes were found to influence the cell‐surface targeting and lysosomal degradation of ABCC1, thereby modulating its efflux capacity. *In vitro* and *in vivo* studies confirmed that C1GALT1 impacts ABCC1 expression and function, further supporting its role in osteosarcoma chemoresistance. These results highlight the clinical relevance of C1GALT1 as a biomarker for prognosis and a potential therapeutic target for osteosarcoma. © 2025 The Author(s). *The Journal of Pathology* published by John Wiley & Sons Ltd on behalf of The Pathological Society of Great Britain and Ireland.

## Introduction

Osteosarcoma (OS) is a primary malignant bone tumor characterized by osteoid and immature bone production, leading to local pain and swelling [1]. In the USA, annual cases range from 750 to 900, while in Taiwan, there are typically 65 to 75 reported cases annually [2, 3]. Although relatively rare, it is the most prevalent bone malignancy in children and adolescents, particularly those aged 10–24 years [4], underscoring its clinical importance. Initial treatment typically involves the so‐called MAP regimen (doxorubicin, cisplatin, and high‐dose methotrexate) combined with limb‐sparing surgery, achieving a 60%–80% 5‐year survival rate for localized disease (662/1,106 and 510/630 patients), while its effectiveness is approximately halved in metastatic cases [5, 6, 7, 8]. OS frequently develops resistance to standard therapies, necessitating improved treatment strategies and the discovery of new therapeutic targets.

Drug resistance poses a significant hurdle in treating OS, driven by genetic mutations, altered drug transport mechanisms, and activated survival pathways in cancer cells [9, 10]. Among these mechanisms, the overexpression of ATP‐binding cassette (ABC) transporters plays a pivotal role by actively expelling chemotherapy drugs from cancer cells, thereby reducing treatment efficacy. Key examples include P‐glycoprotein (P‐gp/ABCB1), multidrug resistance (MDR)‐associated protein 1 (MRP1/ABCC1), and breast cancer resistance protein (BCRP/ABCG2), all of which have been extensively studied in various cancers, including OS [11, 12, 13]. Efforts to develop inhibitors of ABC transporters as chemosensitizers have shown limited clinical success [11]. Here, we propose targeting O‐glycosylation as a novel approach to modulating ABC transporter activity, offering a promising strategy to overcome drug resistance in OS.

Glycosylation, a common posttranslational modification in mammalian cells, plays a crucial role in cancer progression by altering glycosylation patterns [14]. Among various forms of O‐glycosylation, GalNAc‐type O‐glycosylation is predominant, governing numerous functions in both membrane‐bound and secreted proteins [15]. During the synthesis of the GalNAc‐O‐Ser/Thr structure, GALNT family enzymes initiate the transfer of N‐acetylgalactosamine (GalNAc) from UDP‐GalNAc onto serine (Ser) or threonine (Thr) residues. Subsequently, core 1 β1,3‐galactosyltransferase (C1GALT1), the primary core 1 synthase in mammalian cells, catalyzes the transfer of galactose (Gal) from UDP‐Gal to form the Gal‐GalNAc‐O‐Ser/Thr structure, known as the T‐antigen (core 1 structure). This structure serves as the basis for more complex O‐glycans, including the core 2 structure. C1GALT1 is crucial in various biological functions, and its deletion has been linked to developmental defects, spontaneous colitis, and thrombocytopenia in mice [16]. Truncated O‐glycans on cancer cell surfaces are associated with poor outcomes and prognosis in cancer patients. Elevated C1GALT1 levels have been observed in various cancers, including hepatocellular carcinoma, colorectal cancer, breast cancer, head and neck squamous cell carcinoma, prostate cancer, and gastric cancer. This elevation correlates with higher grades, increased recurrence, and reduced survival rates [17]. Given the association between truncated O‐glycans and poor cancer prognosis, we screened for differentially expressed genes (DEGs) in OS and identified the elevated expression of C1GALT1 in poor survival cases and in patients with metastasis within 5 years [18, 19, 20]. However, the role of C1GALT1 in OS remains unclear in clinical studies. At the molecular level, C1GALT1 expression regulates O‐glycosylation of receptors and pathways such as MET in hepatocellular carcinoma, FGFR2 in colorectal cancer, Mucin 1 and CD44 in breast cancer, EGFR and PD‐1 in head and neck squamous cell carcinoma, and EPHA2 in gastric cancer [21, 22, 23, 24], significantly influencing cancer behaviors. This study is the first to explore the mechanism of C1GALT1‐mediated ABC transporter activity in OS drug resistance.

## Materials and methods

### Patients and treatments

This study included tumor biopsy samples from 29 of the initial 92 OS patients, with follow‐up records from 1993 to 2021. Tumor diagnoses were validated histologically from primary tumor specimens before surgery. Data were obtained from National Taiwan University Hospital (NTUH), including clinical parameters such as age, gender, tumor site, and histology. The study was approved by the Institutional Review Board of National Taiwan University Hospital (No. 202401026RINC).

### Immunohistochemistry

Paraffin‐embedded tumor biopsy specimens from 29 NTUH OS patients were used for immunohistochemical (IHC) analysis. To strengthen our findings, we included two additional tissue array cohorts from TissueArray.Com LLC (Derwood, MD, USA): a microarray of 50 patients with survival data (OSCHS801MSur) and a microarray of 63 samples with tumor, node, and metastasis (TNM) staging system and clinical data (OS208a). IHC staining used anti‐C1GALT1 (1:100; clone F‐31, Santa Cruz Biotechnology, Dallas, TX, USA) and anti‐ABCC1 (1:100; clone 1G4A2, Proteintech, Rosemont, IL, USA) antibodies, detected with the Super Sensitive System (BioGenex, Fremont, CA, USA), DAB, and hematoxylin counterstain (Sigma‐Aldrich, St. Louis, MO, USA). IHC results were analyzed using QuPath version 0.4.2 [25], with manual confirmation.

### Accessing the Public Dataset

Gene expression levels of *GALNT1*, *GALNT2*, *C1GALT1*, *GCNT1*, *GCNT3*, and *GCNT4* were evaluated using the R2 Genomics Analysis and Visualization Platform (Amsterdam, North Holland, The Netherlands). This analysis compared expression levels of these O‐linked glycosyltransferases in OS patients with and without metastasis at diagnosis, utilizing data from the Osteosarcoma‐Buddingh‐53‐vst‐ilmnhwg6v2 dataset available in the R2 platform [18].

### Cell lines and cell culture

SaOS‐2, HOS, and G292 cell lines were obtained from the Bioresource Collection and Research Centre (Hsinchu, Taiwan), authenticated via short tandem repeat (STR) profiling. These cells were cultured in Alpha Modification of Eagle's Medium (αMEM, Thermo Fisher Scientific, Waltham, MA, USA) with 10% FBS, penicillin, and streptomycin in a humidified incubator at 37 °C with 5% CO_2_. Mycoplasma contamination screening was conducted regularly using the MycoSEQ™ Mycoplasma Detection Kit (Thermo Fisher Scientific). For specific inhibition of C1GALT1, itraconazole (ITZ) (Sigma‐Aldrich) was added at 2.5 μm and incubated for 3 days, based on preliminary dose–response studies showing effective C1GALT1 inhibition at this concentration (supplementary material, Figure S1). Lentivirus‐based shRNA was used for stable knockdown of *C1GALT1* and ABC transporters (*ABCA3*, *ABCB6*, *ABCC1*, *ABCF1*, *ABCF2*) and *C1GALT1* in pCDH‐EF1‐MCS‐T2A‐Puro for stable overexpression in G292 cells, with clones maintained in 1 μg/ml puromycin (MedchemExpress, Monmouth Junction, NJ, USA). The target sequences of shRNA are listed in supplementary material, Table S1.

### Preparation of doxorubicin‐resistant OS cells

The doxorubicin‐resistant phenotype was induced by gradually exposing cells to increasing concentrations of doxorubicin, up to 512 nm, over approximately 20 weeks. Resistance was confirmed by the absence of cell death and no further resistance increase at higher drug concentrations, alongside the expression of drug resistance‐associated genes (supplementary material, Figure S2).

### ABC transporter expression low‐density array

A custom low‐density array (LDA) was designed to profile the expression of a panel of ABC transporters (Applied Biosystems, Foster City, CA, USA). The LDA plates were preloaded with specific primers and probes for each gene of interest and reference genes (*GAPDH*) for normalization. Reverse transcription and real‐time qPCR was performed using the TaqMan Universal PCR Master Mix (Applied Biosystems) on an Applied Biosystems 7900HT Fast Real‐Time PCR System.

### Quantitative reverse transcription PCR (RT‐qPCR)

Total RNA was extracted with NucleoZOL (MACHEREY‐NAGEL, Düren, North Rhine‐Westphalia, Germany), and 1 μg was used for cDNA synthesis with a Moloney murine leukemia virus reverse transcription kit (Protech Technology Enterprise, Taipei, Taiwan). Gene expression was quantified using SYBR Green qPCR (Protech Technology Enterprise). The primers used are listed in supplementary material, Table S1.

### Flow cytometry analysis of lectin binding and cellular apoptosis

Cells (1 × 10^5^) were suspended in 100 μl PBS with 0.5% BSA, treated with neuraminidase (Sigma‐Aldrich), and incubated with Vicia villosa agglutinin (VVA) lectin‐FITC (Vector Laboratories, Newark, CA, USA) to detect O‐glycan changes and Annexin V‐FITC (Bio Pioneer Tech Co., New Taipei City, Taiwan) to assess apoptosis. After washing, the fluorescence intensity of 10,000 cells was analyzed using flow cytometry (BD Pharmingen, Franklin Lakes, NJ, USA). Negative controls included samples without staining. Data represent three independent experiments.

### Western blot analysis and lectin pull‐down assay

Cell lysates were prepared in lysis buffer (Thermo Fisher Scientific), and proteins were separated on 10% SDS‐PAGE gels and transferred onto PVDF membranes. Membranes were blocked with 5% BSA (Bio‐Rad, Hercules, CA, USA) in Tris‐buffered saline with Tween 20 and probed with antibodies against C1GALT1 (1:1,000; Santa Cruz Biotechnology), ABCC1 (1:1,000; Proteintech), and GAPDH (1:1,000; Santa Cruz Biotechnology). Detection used HRP‐conjugated secondary antibodies and ECL reagents (GE Healthcare, Chicago, IL, USA). For lectin pull‐down assays, 500 μg of lysate proteins were incubated with VVA lectin‐conjugated beads (Vector Laboratories) and analyzed by SDS‐PAGE for western blotting.

### Immunofluorescence staining

Cells were cultured on chamber slides (SPL Life Sciences, Pocheon‐si, South Korea), fixed with 4% paraformaldehyde, permeabilized with 0.25% Triton X100, and blocked with 2% BSA (Bio‐Rad). They were incubated with primary antibodies for ABCC1 (1:200; Proteintech) and GM130 (1:500; GTX130351GeneTex, Irvine, CA, USA), followed by Alexa Fluor secondary antibodies (Thermo Fisher Scientific). Nuclei were counterstained with DAPI (Santa Cruz Biotechnology). Images were captured on a Carl Zeiss LSM880 confocal microscope (Oberkochen, Baden‐Württemberg, Germany) as single planes.

### Time‐lapse doxorubicin efflux assay

To ensure doxorubicin entry, cells on coverslips were treated with 10 μm doxorubicin (MedchemExpress) for 2 h, then washed with PBS. Doxorubicin autofluorescence (Ex/Em = 488/600 nm) was monitored using total internal reflection fluorescence (TIRF)/spinning disk confocal microscopy (Carl Zeiss, TIRF 3/Cell Observer SD; Imaging Core, First Core Labs, NTU College of Medicine). FIJI (ImageJ; NIH, Bethesda, MD, USA) was used to quantify fluorescence intensity in regions of interest.

### Animals

Female NOD‐SCID mice (six per group), from the National Laboratory Animal Center, Taipei, were housed in a pathogen‐free facility with controlled conditions under Institutional Animal Care and Use Committee approval (No.: 20230361, NTU College of Medicine). G292 and HOS cells with different transfectants (5 × 10^6^) were injected bilaterally into tibiae to study drug resistance and tumor growth. Ten days after injection, doxorubicin (in DMSO) was administered every 3 days at 10 mg/kg i.p., with tumor growth monitored using In Vivo Imaging System (IVIS), (PerkinElmer Taiwan Corporation, Taipei, Taiwan) and calipers. Mice were euthanized on day 30 for evaluation.

### Statistical analyses

Statistical analyses were conducted using SAS enterprise guide 8.3 for Windows software. Associations between pairs of categorical variables were assessed using Pearson's 𝜒^2^ test. Survival probabilities in various subgroups were estimated using the Kaplan–Meier method and analyzed using log‐rank tests. The influence of each variable on survival was assessed using the univariate and multivariate Cox proportional hazard model. The significance of the variations between the data resulting from different experiments was analyzed using Student's *t*‐test. All statistical tests were two sided, and those with a *p* value <0.05 were considered to be statistically significant.

## Results

### High‐level expression of C1GALT1 protein is associated with poorly differentiated OS tumors, signifying adverse survival and recurrence trends

**Figure 1:**
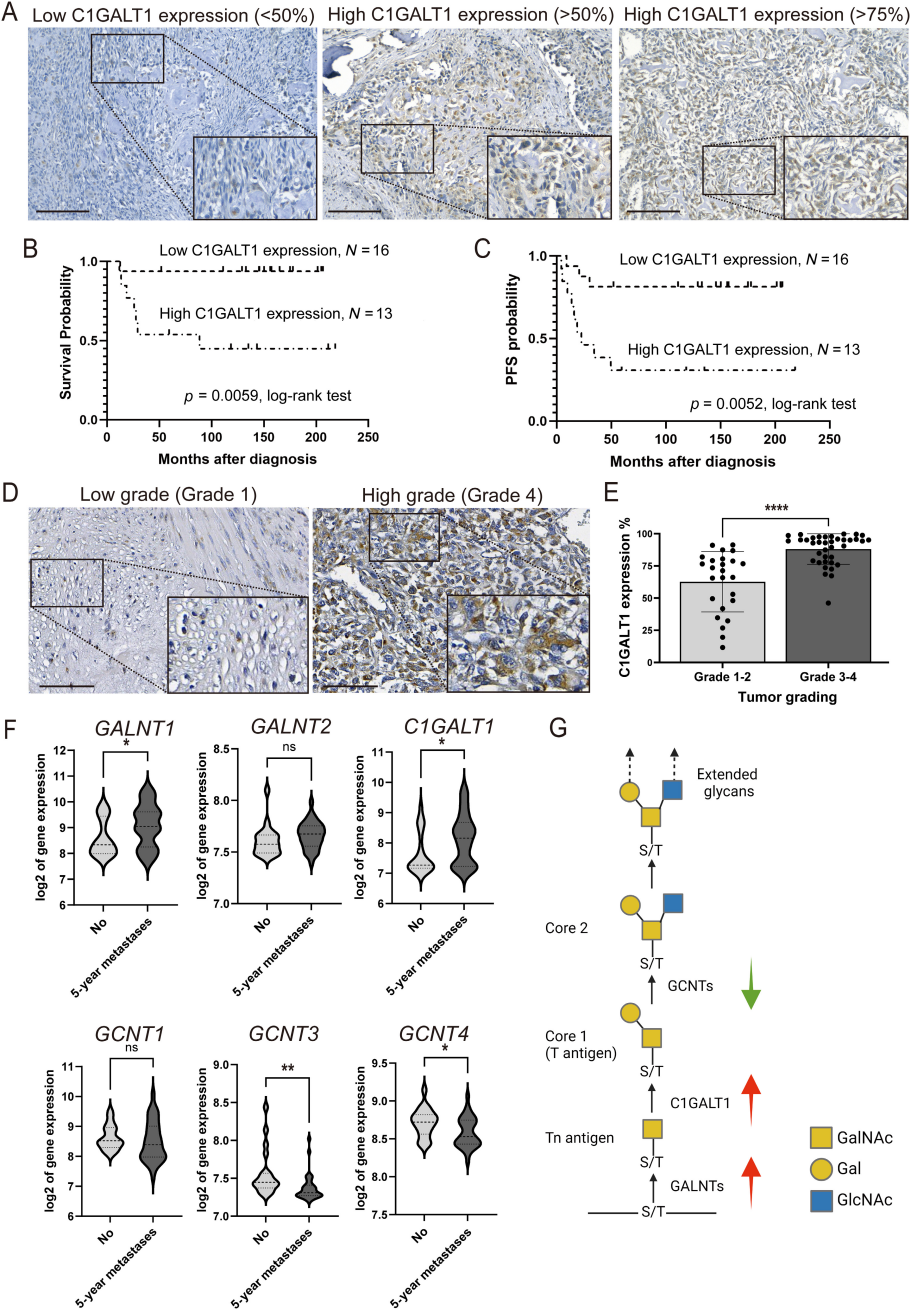
C1GALT1 expression predicts poor prognosis in OS patients. (A) Representative IHC images showing C1GALT1 protein staining in OS tumors. Brown color indicates positive staining. Scale bars, 100 μm; high‐magnification images of marked area are shown in lower right corner. (B and C) Kaplan–Meier curves depicting overall survival and PFS according to C1GALT1 expression in 29 OS patients (*p* = 0.0059 and 0.0052, respectively, log‐rank test). (D) Representative IHC images showing C1GALT1 protein staining in low‐grade and high‐grade OS tumors from the tissue array (OS208a). Scale bars, 100 μm; high‐magnification images of marked area. (E) Scatter plot with bars representing C1GALT1 expression percentages among 63 OS samples from tissue array (OS208a). Student's *t*‐test was used to compare expression levels between low‐ and high‐grade tumors (****p < 0.0001). (F) Violin plots illustrating RNA‐level expressions of related O‐glycosyltransferase genes in patients with or without 5‐year metastasis, using the ilmnhwg6v2 dataset from R2. Log2: gene expression transformed into logarithm with base 2. (G) Visualization of relative functions of O‐glycosyltransferases influencing major O‐glycan structures, based on dataset. This figure was created with BioRender.com.

In this study, we employed IHC staining to examine C1GALT1 protein expression in a subset of 29 patients with OS tumors obtained from NTUH. Positive C1GALT1 staining was observed in the Golgi apparatus of OS cells. Using QuPath version 4.2 with manual confirmation [25], cells were classified based on C1GALT1 expression (<50% or ≥50%), grouping patients accordingly (Figure 1A). Table 1 shows no notable differences in most clinicopathological characteristics such as sex, age at diagnosis, or serum alkaline phosphatase (ALP) or lactate dehydrogenase (LDH) levels between the groups with high and low C1GALT1 expression. However, the high‐expression group showed significantly higher rates of recurrence and mortality. Kaplan–Meier analysis further highlighted that patients with high C1GALT1 expression experienced markedly worse overall survival and progression‐free survival (PFS) outcomes compared to those with low C1GALT1 expression (Figure 1B,C and Table 2).

**Table 1:** C1GALT1 expression and clinicopathological and biological characteristics of osteosarcoma (OS) patients.

Characteristics	All patients (*N* = 29)	C1GALT1 low expression (*N* = 16)	C1GALT1 high expression (*N* = 13)	*p* value
Age at diagnosis (years)	13.03 ± 2.72 (6.00–17.10)	13.13 ± 2.34	12.92 ± 3.14	0.8395
Sex				0.3787
Male	16 (55.17%)	10	6	
Female	13 (44.83%)	6	7	
Primary location				0.8202
Femur	18 (62.07%)	10	8	
Tibia	6 (20.69%)	4	2	
Humerus	3 (10.34%)	1	2	
Others	2 (6.90%)	1	1	
Location within long bone				0.4503
Distal	18 (62.07%)	11	7	
Proximal	10 (34.48%)	5	5	
Serum ALP at diagnosis	681.31 ± 580.82 (106.00–2,266.00)	639.50 ± 242.87	732.77 ± 671.19	0.6842
Serum LDH at diagnosis	513.71 ± 252.15 (173.00–1,173.00)	482.56 ± 242.87	555.25 ± 268.93	0.4685
Histological type				0.8153
Osteoblastic	21 (72.41%)	11	10	
Chondroblastic	2 (6.90%)	1	1	
Others	6 (20.69%)	4	2	
Operation type				0.2589
Amputation	28 (96.55%)	16	12	
Limb‐sparing surgery	1 (3.45%)	0	1	
Recurrent after therapy				**0.0061** ^**^
Yes	12 (41.38%)	3	9	
No	17 (58.62%)	13	4	
Overall survival (until last follow‐up)				**0.0043** ^***^
Survive	21 (72.41%)	15	6	
Expired	8 (27.59%)	1	7	

*Note*: Bold indicates statistically significant **p < 0.01, ***p < 0.005.

**Table 2:** Clinicopathological and biological factors affecting survival rate and recurrent rate in OS patients.

Variable	Univariable OS analysis			Univariable PFS analysis		
	RR	95% CI	*p*	RR	95% CI	*p*
Age at diagnosis (years)	1.364	0.923–2.017	0.1192	1.131	0.845–1.515	0.4082
Serum ALP at diagnosis	1.001	1.000–1.002	0.1549	1.001	0.999–1.002	0.3425
Serum LDH at diagnosis	1.001	0.998–1.004	0.4895	1.000	0.996–1.003	0.7540
C1GALT1 expression level (≥50%)	17.499	1.756–174.405	0.0147*	9.750	1.743–54.523	**0.0095** ^**^

*Note*: Bold indicates statistically significant; *p < 0.05, **p < 0.01.

Given the limitation of a relatively small number of available tumor biopsy samples (29 out of the initial 92 patients), we confirmed that this biopsy cohort was representative of the original patient pool, with no significant differences in most clinical characteristics (supplementary material, Table S2). To further strengthen our findings, we included two additional tissue array cohorts: one comprising 50 OS patients with survival data (OSCHS801MSur, supplementary material, Figure S3), which produced results consistent with those observed in the NTUH cohort, confirming the link between high C1GALT1 expression and poor survival. The second cohort included 63 OS patients with TNM staging and clinical stage data (OS208a), which allowed us to analyze the association between C1GALT1 expression and tumor grade. Greater C1GALT1 expression (75% of cells stained) was notably observed in high‐stage tumors (Figure 1D). Statistical analysis revealed that elevated C1GALT1 expression was present in 60.3% (38/63) of high‐grade OS tumors (Figure 1E), indicating a significant association between high C1GALT1 expression and aggressive OS tumors. To validate our findings on C1GALT1 expression in OS tumors, we utilized the ilmnhwg6v2 dataset from R2, ‘Genomics Analysis and Visualization Platform’, as independent cohorts [18]. The analysis showed a significant increase in *GALNT1* (polypeptide N‐acetylgalactosaminyltransferase 1) and *C1GALT1* expression in 5‐year metastatic OS patients compared to nonmetastatic patients (*p* = 0.036 and 0.033, respectively). However, *GCNT3* and *GCNT4* (glucosaminyl (N‐acetyl) transferase), which encode the core 2 O‐glycan branching enzyme, were downregulated in 5‐year metastatic OS patients (Figure 1F). The relative functions of O‐glycosyltransferases, influencing the main O‐glycan structures, are visually depicted in Figure 1G. These results suggest that C1GALT1 plays a pivotal role in shaping the O‐glycosylation pattern in OS, highlighting its clinical significance in predicting adverse survival outcomes in patients.

### C1GALT1 expression is elevated in doxorubicin‐selected OS cells, and its suppression promotes doxorubicin‐induced apoptosis while hampering doxorubicin efflux

**Figure 2:**
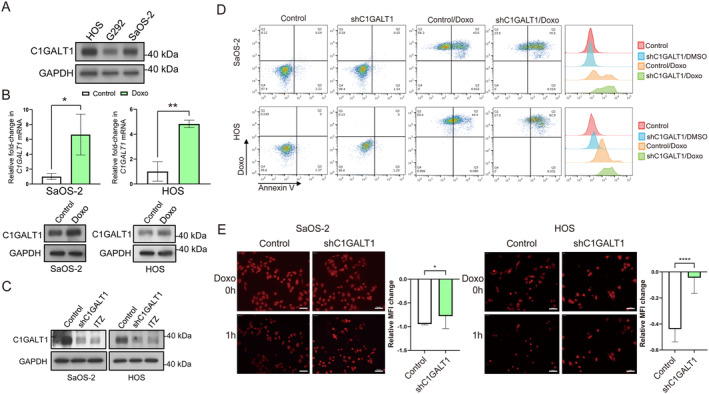
Targeting C1GALT1 promotes doxorubicin‐induced apoptosis while impairing doxorubicin efflux. (A) Immunoblotting analysis for C1GALT1 expression in various OS cell lines, using GAPDH as an internal control. (B) RT‐qPCR analysis showing mRNA expression levels of *C1GALT1* in SaOS‐2 and HOS cells stimulated with 50 nm doxorubicin for 48 h, using *ACTB* as an internal control. Immunoblotting analysis for C1GALT1 and GAPDH protein expression levels in the same treated cells. (C) Immunoblotting analysis of C1GALT1 expression in SaOS‐2 and HOS OS cell lines transfected with C1GALT1 shRNA or treated with itraconazole (2.5 μm), using GAPDH as an internal control. (D) Representative flow cytometry plots showing percentages of apoptotic cells in doxorubicin‐treated cells transfected with C1GALT1 shRNA or scramble control. Overlay histogram plots are shown on the right‐hand side of the panel. (E) Representative fluorescence images taken by TIRF/confocal microscope display residual intensity of doxorubicin (red) in cells over 1 h. Scale bar, 50 μm. *p < 0.05, ****p < 0.0001. Bars represent mean fluorescence intensity changes of randomly selected cells (*n* = 30).

To investigate the role of C1GALT1 in OS drug resistance, we analyzed doxorubicin‐induced apoptosis and drug efflux assays using a variety of cell lines with different malignant phenotypes [26]. Specifically, western blot analysis initially showed elevated C1GALT1 protein levels in highly aggressive cell lines HOS and SaOS‐2 compared to the less aggressive G292 cell line (Figure 2A). Additionally, both mRNA and protein expression levels of C1GALT1 were elevated in doxorubicin‐selected cells (Figure 2B). To assess the impact of C1GALT1 on OS doxorubicin resistance, we stably transfected HOS and SaOS‐2 cells with shC1GALT1 or control lentivirus. Western blotting confirmed the efficiency of silencing, as shown in Figure 2C. Flow cytometry demonstrated that the knockdown of *C1GALT1* resulted in a 33% increase in apoptotic cells upon doxorubicin challenge (Figure 2D). Moreover, chemotherapy resistance is significantly influenced by multidrug efflux pumps. Traditional methods, such as endpoint fluorescent analysis through flow cytometry, employed to monitor transporter efflux activity may have limitations, particularly in providing real‐time evidence. In this investigation, we explored the use of TIRF/spinning disk confocal microscopy to observe drug efflux mediated by a multidrug transporter. Representative images illustrated that C1GALT1‐silenced cells exhibited a reduction in doxorubicin efflux compared to the control group, manifested as losses of fluorescence intensity occurring at different time points after doxorubicin stimulation. The quantification of changes in fluorescent intensity is presented in the accompanying panel (Figure 2E). These findings indicate that the suppression of C1GALT1 enhances doxorubicin‐induced apoptosis while impeding doxorubicin efflux.

### Itraconazole‐mediated inhibition of C1GALT1 mirrors the effects of enhancing doxorubicin‐induced apoptosis and impairing doxorubicin efflux

**Figure 3:**
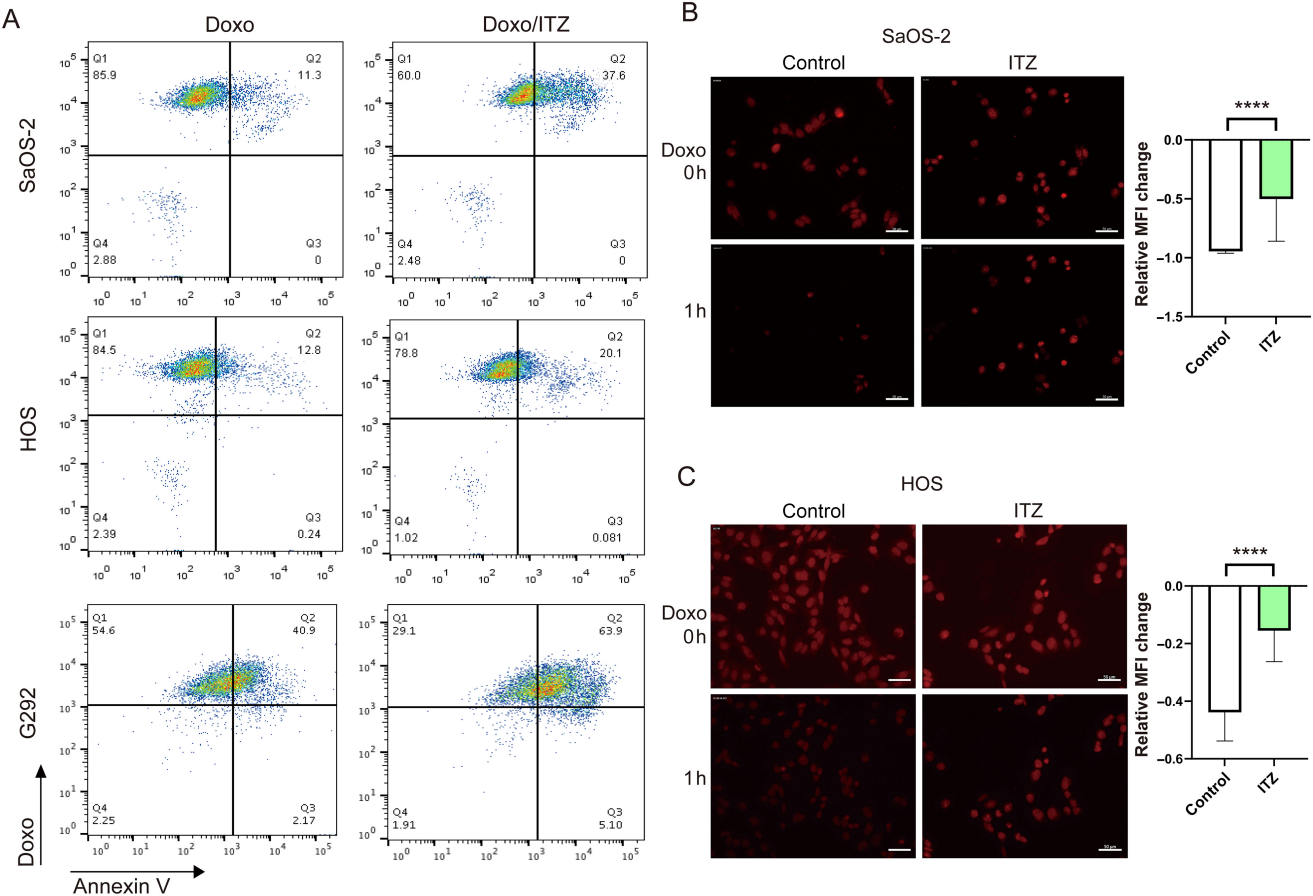
ITZ mimics the effects of boosting doxorubicin‐induced apoptosis and reducing doxorubicin efflux. (A) Representative flow cytometry plots showing percentages of apoptotic cells in doxorubicin (50 nm) or ITZ) (2.5 μm)‐treated OS cells. (B and C) Representative fluorescence images taken by TIRF/confocal microscope display residual intensity of doxorubicin (red) in cells over 1 h. Scale bar, 50 μm. ****p < 0.0001. Bars represent the mean fluorescence intensity changes of randomly selected cells (*n* = 30).

Repurposing old drugs for oncological purposes offers a cost‐effective approach to cancer treatment. ITZ, a triazole antifungal, has shown clinical activity in oncology, with trials demonstrating efficacy in prostate, lung, and basal cell carcinoma treatment combinations. Reports also suggest potential activity against leukemia, ovarian, breast, and pancreatic cancers [27]. Azole antifungals like ITZ are known to inhibit drug resistance through P‐glycoprotein (P‐gp/ABCB1) and BCRP [28, 29]. However, ITZ's specific anticancer impact in OS remains unclear, including its role in P‐gp and BCRP‐mediated efflux mechanisms. Lin *et al* discovered a new role for ITZ in inhibiting C1GALT1 by promoting proteasome degradation [30]. In the subsequent investigation, our aim was to assess the effects of ITZ on doxorubicin‐induced apoptosis and drug efflux in OS cells. Western blotting confirmed a reduction in C1GALT1 protein expression levels in ITZ‐treated cells (Figure 2C). Flow cytometry demonstrated a similar trend to *C1GALT1* knockdown, indicating that ITZ‐mediated inhibition of C1GALT1 resulted in an increase in apoptotic cells upon doxorubicin challenge (Figure 3A). Representative images from TIRF/spinning disk confocal microscopy illustrated that ITZ‐treated cells exhibited a reduction in doxorubicin efflux compared to the control group (Figure 3B,C). The quantification of changes in fluorescence intensity is presented in the accompanying panel. These results indicate that the pharmacological inhibition of C1GALT1 enhances doxorubicin‐induced apoptosis while impeding doxorubicin efflux, emphasizing ITZ's potential in treating OS drug resistance.

### Identification of ABCC1 in doxorubicin resistance and efflux and its clinical correlation to C1GALT1 in OS

**Figure 4:**
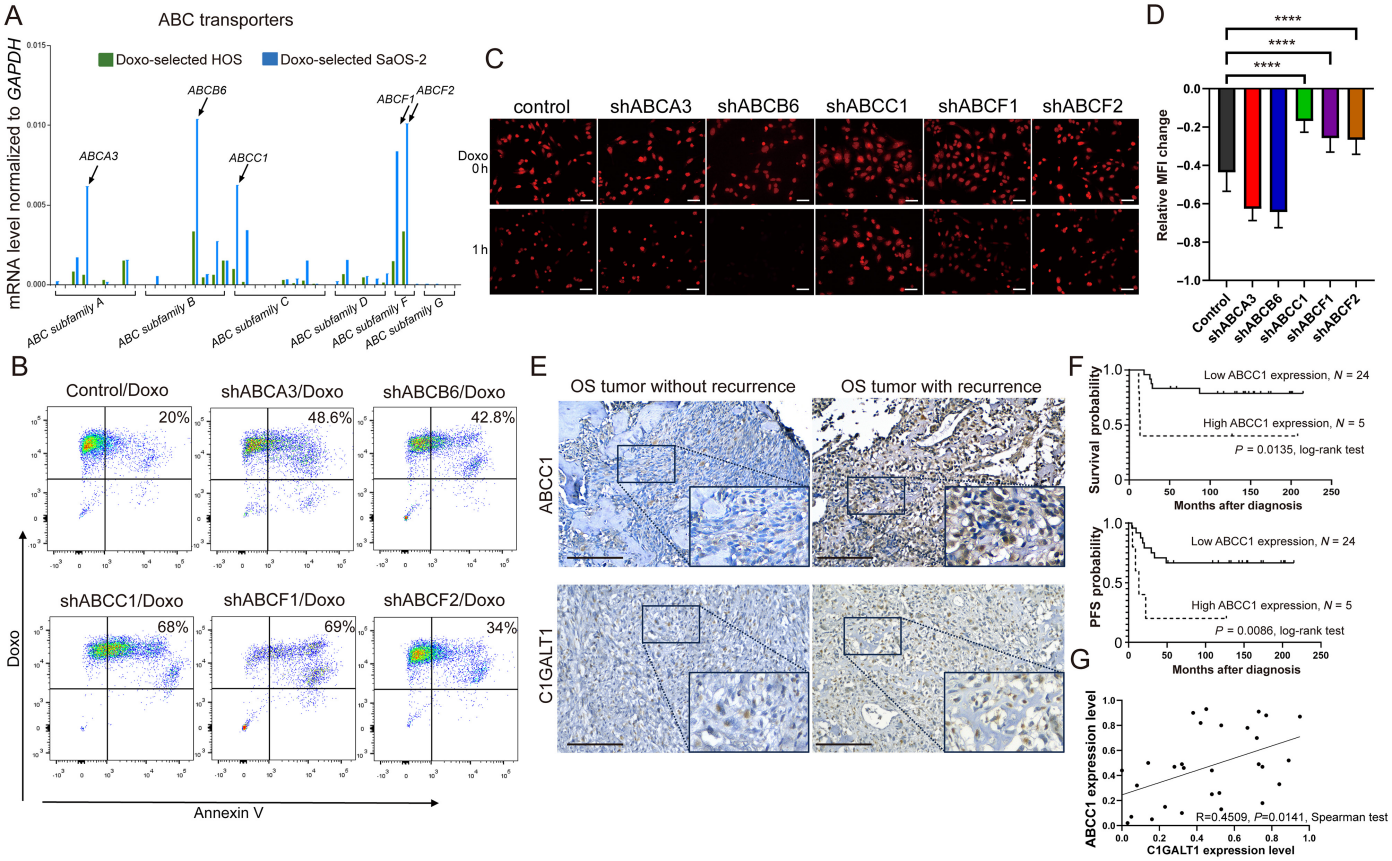
ABCC1 identification in doxorubicin resistance and efflux and clinical correlation to C1GALT1 in OS. (A) Bar charts depict ABC transporter gene expression using the TaqMan™ Human ABC Transporter Array in doxorubicin‐selected HOS and SaOS‐2 cell lines, covering 50 genes across seven ABC transporter families. Arrows indicate the relatively high expression of ABC transporters, with *GAPDH* used as an internal control. (B) Representative flow cytometry plots show percentages of apoptotic cells in doxorubicin‐treated cells transfected with corresponding ABC shRNA or scramble control. (C and D) Representative fluorescence images captured by TIRF/confocal microscopy demonstrate residual intensity of doxorubicin (red) in cells over 1 h. Scale bar, 50 μm. Bars represent mean fluorescence intensity changes of randomly selected cells (*n* = 30). ****p < 0.0001. (E) Representative IHC images display C1GALT1 and ABCC1 protein staining in OS tumors with and without recurrence. Brown color indicates positive staining. Scale bars, 100 μm; high‐magnification images of marked area are shown in lower right corner. (F) Kaplan–Meier curves depict overall survival and PFS according to ABCC1 expression in 29 OS patients (*p* = 0.0135 and 0.0086, respectively, log‐rank test). (G) Pearson correlation analysis of C1GALT1 and ABCC1 protein expression in the same patient cohort (*r* = 0.45, *p* < 0.05).

MDR poses a significant challenge in cancer treatment by reducing the effectiveness of chemotherapeutic agents like doxorubicin. ATP‐binding cassette (ABC) transporters, which actively pump drugs out of cancer cells, contribute significantly to this resistance [31]. Our study aimed to explore the relationship between C1GALT1 and ABC transporters in doxorubicin‐selected OS cells, offering potential insights for novel therapeutic strategies. To assess this, we analyzed samples from doxorubicin‐selected HOS and SaOS‐2 cell lines using a human ABC transporter array, which included probes for 50 genes across seven ABC families. Seventeen genes were consistently detected in both cell lines, with *ABCA3*, *ABCB6*, *ABCC1*, *ABCF1*, and *ABCF2* exhibiting the highest expression levels (mean Ct<30) (Figure 4A). Subsequently, we evaluated the role of these ABC transporters in doxorubicin drug resistance and efflux. RT‐qPCR confirmed a reduction in mRNA expression levels for ABC transporters in cells transfected with shRNA targeting *ABCA3*, *ABCB6*, *ABCC1*, *ABCF1*, and *ABCF2* (supplementary material, Figure S4). Flow cytometry revealed that knockdown of *ABCC1* significantly increased the sensitivity of OS cells with 48% to doxorubicin treatment (Figure 4B). TIRF confocal microscopy and quantitative analysis confirmed a more than 50% increase in fluorescence intensity accumulation upon *ABCC1* silencing compared to controls, indicating minimal reduction in doxorubicin accumulation across all groups (Figure 4C,D).

To further validate our findings, we examined ABCC1 protein expression in a subset of 29 OS patients using tumor samples obtained from NTUH, employing IHC staining (Figure 4E). Kaplan–Meier analysis revealed that patients with high ABCC1 expression experienced significantly worse overall survival and PFS compared to those with low ABCC1 expression (Figure 4F). Additionally, we analyzed the correlation between C1GALT1 and ABCC1 protein expression in the same subset of patients. Pearson correlation analysis showed that C1GALT1 expression was positively correlated with ABCC1 protein expression (*r* = 0.45, *p* < 0.05) (Figure 4G). These results prompted us to investigate whether ABCC1 is involved in drug resistance mediated by C1GALT1.

### ABCC1 is involved in phenotypic changes mediated by C1GALT1 in OS cells both *in vitro* and *in vivo*

**Figure 5:**
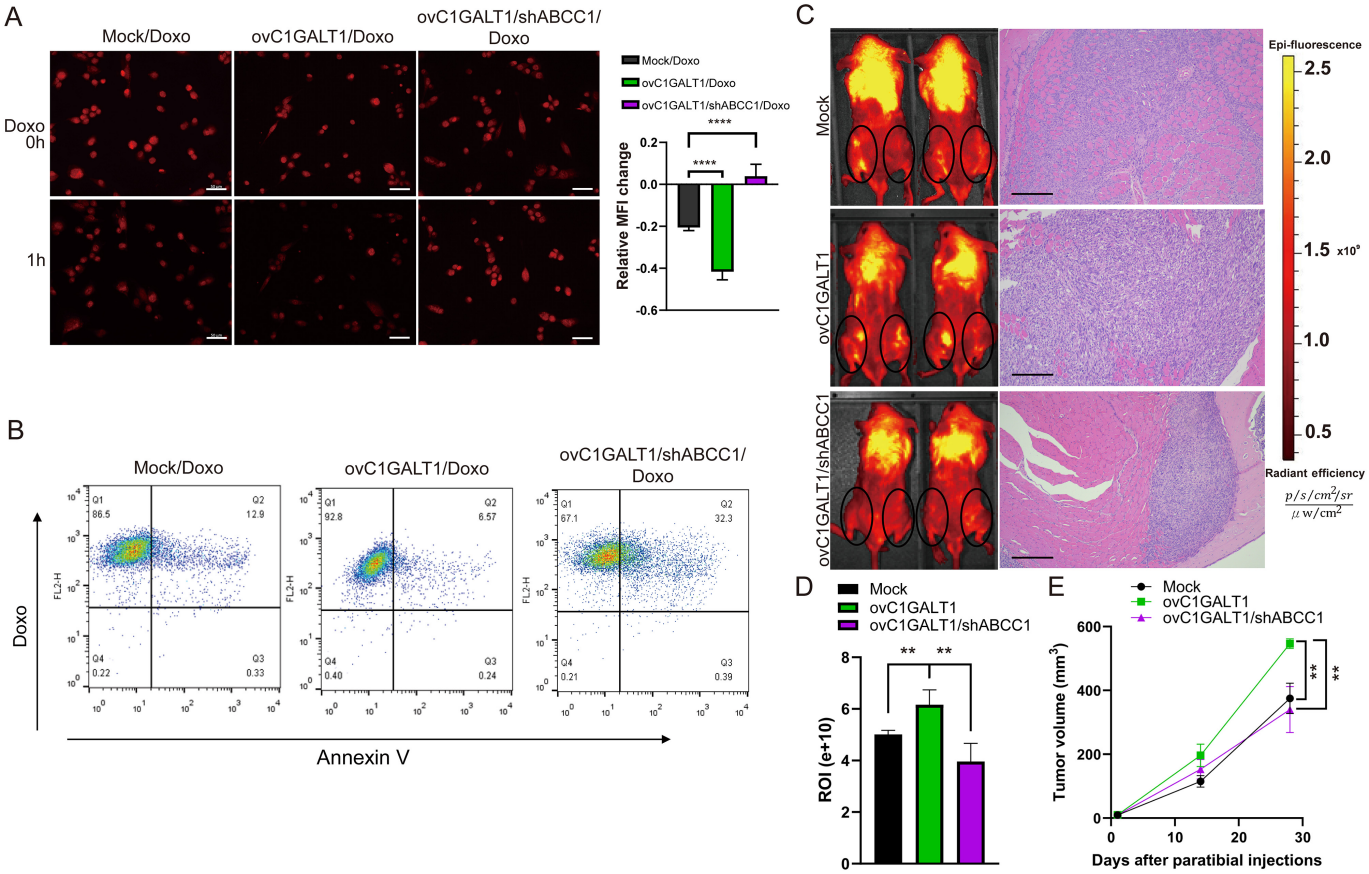
ABCC1 is involved in the phenotypic changes mediated by C1GALT1 in OS cells both *in vitro* and *in vivo*. (A) Fluorescence images captured using TIRF/confocal microscopy display the residual intensity of doxorubicin (red) in mock, C1GALT1‐overexpressing, and C1GALT1‐overexpressing with ABCC1‐silenced cells over 1 h. Scale bar, 50 μm. Bars represent mean fluorescence intensity changes of randomly selected cells (*n* = 30). ****p < 0.0001. (B) Representative flow cytometry plots display percentages of apoptotic cells in doxorubicin‐treated samples. (C) IVIS images of representative mice taken at 2‐week intervals, highlighting the regions of interest (ROIs, marked by ovals) around tibial tumors in mice injected with either G292 mock‐transfected cells (black bar and circles), C1GALT1‐overexpressing cells (ovC1GALT1, green bar and squares), or *C1GALT1*‐overexpressing cells with *ABCC1* knockdown (ovC1GALT1/shABCC1, purple bar and triangles). H&E‐stained sections of tibial tumors are shown on the right. (D) Bars represent mean bioluminescent signal intensity within tumor ROIs (*n* = 6). **p < 0.01. Tumor growth curves show tumor volume (mm^3^). (E) Tumor volumes were measured by calipers at 0, 14, and 28 days after injection. *p* < 0.01 at 28 days, indicating significant differences between ovC1GALT1 group and both the mock and ovC1GALT1/shABCC1 groups.

We investigated whether C1GALT1 contributed to drug resistance and tumor growth in OS cells through the ABCC1 pathway. Western blotting confirmed the protein levels of C1GALT1 and ABCC1 in cells overexpressing *C1GALT1* and in cells with *C1GALT1* overexpression combined with *ABCC1* silencing (supplementary material, Figure S5). We examined the impact of *C1GALT1* overexpression on drug resistance by treating OS cells with doxorubicin and assessing drug efflux and apoptosis. Additionally, we evaluated whether *ABCC1* silencing could reverse *C1GALT1*‐mediated drug resistance and related phenotypic changes. TIRF confocal microscopy indicated that cells with *ABCC1* silencing reversed the reduction in doxorubicin efflux compared to *C1GALT1* overexpressing groups (Figure 5A). The quantification of changes in fluorescence intensity is presented in the accompanying panel. Flow cytometry confirmed that knocking down *ABCC1* increased the sensitivity of *C1GALT1* overexpressing OS cells to doxorubicin treatment by 26% (Figure 5B). We validated our findings using a xenograft mouse model of human OS via paratibial injection. G292 cells with either *C1GALT1* overexpression or *ABCC1* knockdown were implanted in immunodeficient mice. Tumor growth was monitored following doxorubicin treatment, assessed by IVIS imaging, and confirmed with H&E staining. *ABCC1* knockdown reversed *C1GALT1*‐mediated tumor growth by 36% in response to doxorubicin challenge (Figure 5C,D). By day 28, tumor volume analyzed by calipers indicated a 46% increase in the *C1GALT1* overexpression group compared to the control group, while *ABCC1* knockdown reduced tumor growth by 38%, aligning with the reduction observed in the IVIS analysis (Figure 5E). Our findings indicated that C1GALT1 significantly enhanced drug resistance and tumor growth in OS cells, primarily through the ABCC1 pathway.

### O‐glycosylation changes induced by silencing C1GALT1 hinder ABCC1 cell‐surface targeting and promote its lysosomal degradation

**Figure 6:**
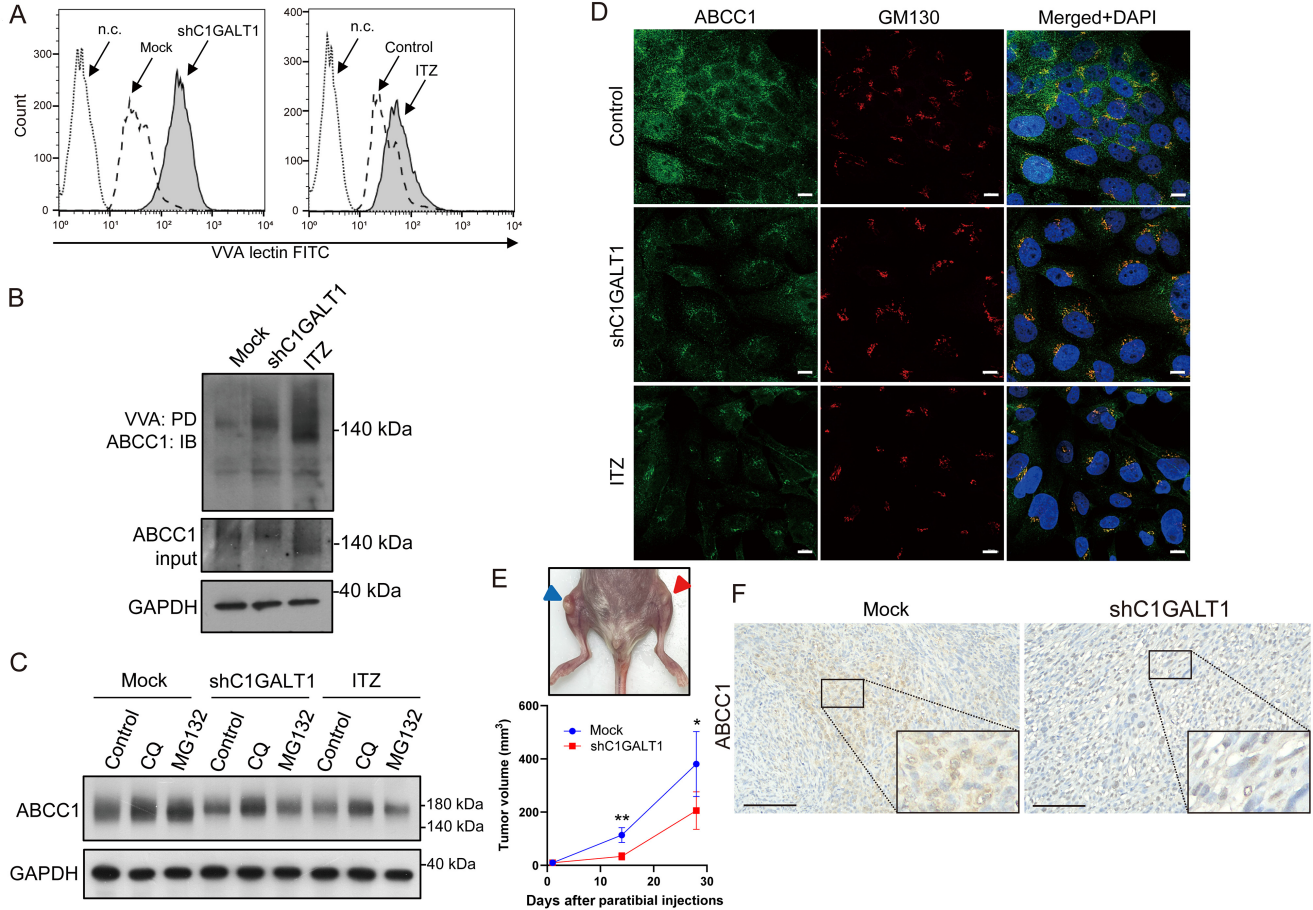
O‐glycosylation changes induced by silencing C1GALT1 hinder ABCC1 cell‐surface targeting and promote its lysosomal degradation. (A) Histograms showing VVA lectin binding fluorescence intensity in mock, C1GALT1‐silenced, and itraconazole‐treated cells. HOS cells were stained with FITC‐conjugated VVA lectin or without lectin (n.c.). (B) Western blot showing proteins pulled down (PD) by VVA lectin in mock, C1GALT1‐silenced, and itraconazole‐treated cells, then immunoblotted (IB) with anti‐ABCC1. GAPDH was the internal control. (C) Western blot demonstrating ABCC1 expression in HOS cells treated with CQ and MG132 inhibitors (10 μm each). (D) Immunofluorescence staining of mock, C1GALT1‐silenced, and itraconazole‐treated cells for ABCC1 (green) and GM130 (red). Colocalization at the Golgi apparatus is shown in yellow in the merged image of C1GALT1‐silenced samples. (E) Representative image showing tumors from paratibial injection of HOS cells in mice. Blue arrowheads indicate mock group; red arrowheads indicate the *C1GALT1* knockdown group. Tumor volume, calculated as (length × width^2^)/2, is plotted over time (*n* = 6). *p < 0.05, **p < 0.01. (F) IHC images showing ABCC1 staining in tibia tumors. Brown indicates positive staining. Scale bars, 100 μm. High‐magnification images of marked areas are in lower right corner.

Several studies have highlighted the crucial role of C1GALT1‐mediated O‐glycosylation in regulating the surface localization and stability of transmembrane glycoproteins such as MUC1, CD44, and IL‐6 receptor, thereby impacting tumor growth and metastasis [21, 24]. However, its influence on ABC transporters, which are involved in diverse cellular processes including drug resistance, remains unclear in OS. We investigated the impact of manipulating C1GALT1 expression on O‐glycosylation in OS cells. Flow cytometry revealed enhanced VVA lectin binding to cell surfaces upon reducing C1GALT1 through shRNA or ITZ treatment (Figure 6A). Lectin pull‐down and immunoblotting assays confirmed increased ABCC1 binding to VVA lectin beads upon C1GALT1 inhibition, suggesting the presence of O‐linked glycans on ABCC1 (Figure 6B). We conducted western blotting and immunofluorescence staining to assess ABCC1 expression and localization in C1GALT1‐inhibited cells. Western blot results demonstrated reduced ABCC1 expression in C1GALT1‐inhibited OS cells. Treatment with chloroquine, but not MG132, reversed ABCC1 expression, indicating that C1GALT1 inhibition primarily reduced ABCC1 through the lysosomal degradation pathway (Figure 6C). In addition, confocal microscopy revealed that in C1GALT1‐inhibited cells, ABCC1 was predominantly localized in the Golgi apparatus, colocalizing with the GM130 marker (Figure 6D). To confirm whether the truncation of O‐glycans by silencing C1GALT1 affected cell‐surface targeting of ABCC1 *in vivo*, we injected immunodeficient mice with mock or *C1GALT1* knockdown OS cells via paratibial injection. The results demonstrated that *C1GALT1* knockdown reduced tumor nodules in the tibia region, consistent with tumor volume analysis (Figure 6E). Furthermore, IHC staining revealed that ABCC1 was predominantly localized in the Golgi apparatus of *C1GALT1* knockdown tumor cells (Figure 6F), consistent with our *in vitro* findings. In conclusion, this study highlights the critical role of C1GALT1‐mediated O‐glycosylation in regulating the surface localization and stability of ABCC1, suggesting potential implications for cancer drug resistance.

## Discussion

This study explored how C1GALT1 drives chemoresistance in OS. Elevated C1GALT1 levels are linked to advanced stages, poor survival, and recurrence. In doxorubicin‐treated cells, C1GALT1 enhances resistance by promoting efflux, which can be mitigated by C1GALT1 suppression or ITZ. ABCC1 is identified as a key mediator, with C1GALT1 affecting its surface targeting and degradation through O‐glycan modifications. These findings highlight C1GALT1's role in OS chemoresistance via ABCC1 regulation.

C1GALT1 has garnered attention in oncology due to its role in tumor development. Elevated C1GALT1 expression is associated with poorer prognoses in cancers including those of the head and neck, breast, liver, colon, ovarian, and pancreatic. However, in neuroblastoma, higher C1GALT1 expression correlates with better outcomes, highlighting the need to further investigate its role in OS [30, 32, 33, 34]. Watanabe *et al* also identified C1GALT1 as one of the upregulated genes in poor prognosis clusters from two independent OS cohorts in the Gene Expression Omnibus (GEO) dataset [35].

Using these same datasets, we conducted an independent analysis to validate and expand upon these findings. Our analysis further confirmed elevated C1GALT1 expression in poor survival cases and in patients who developed metastasis within 5 years, thereby reinforcing the association between high C1GALT1 expression and adverse outcomes in OS (supplementary material, Figure S6). Our investigation is distinguished by its in‐depth analysis across a local NTUH cohort and two OS tissue array cohorts, revealing significant C1GALT1 overexpression in advanced‐stage OS, associated with poor survival, and tumor recurrence. Elevated C1GALT1 also emerged as an independent prognostic marker for poor survival in OS patients.

Drug resistance is a major obstacle in treating cancer recurrence. Initial successes with chemotherapeutics like doxorubicin, cisplatin, and methotrexate have been overshadowed by emerging resistance, leading to relapse [36]. Classical acquired MDR often involves reduced drug accumulation mediated by ABC transporters such as P‐gp (ABCB1), BCRP (ABCG2), and MRP1 (ABCC1) from the ABC protein superfamily, recognized for conserved sequences in their nucleotide‐binding domain. Our research highlights the critical role of ABCC1 in OS drug resistance, identified through ABC array screening and validated by *in vitro* and *in vivo* experiments, showing ABCC1's significant impact on doxorubicin efflux and treatment sensitivity. Furthermore, this study is the first to establish a clinical correlation between high ABCC1 expression in OS patients and poor survival outcomes.

Among these pathways, considerable research has focused on N‐glycan modifications linked to drug resistance [37, 38]. However, the impact of O‐glycan modifications remains uncertain. For instance, GALNT14 is essential for P‐gp stability on the plasma membrane in breast cancer and is correlated with oxaliplatin resistance in colorectal cancer [39, 40]. Building on ABCC1's role in drug resistance, our study shows that disrupting C1GALT1‐mediated O‐glycosylation, genetically or pharmacologically, leads to significant Golgi retention of ABCC1, impairing its cell‐surface expression and hindering doxorubicin efflux. Supporting this, C1GALT1 overexpression enhances ABCC1 plasma membrane localization (supplementary material, Figure S7). These *in vitro* findings align with *in vivo* observations in which IHC staining revealed perinuclear ABCC1 in C1GALT1‐deficient OS cells.

Our results align with studies indicating that ABCC1 mislocalization increases chemotherapy sensitivity in cisplatin‐ and venetoclax‐resistant cancers [41, 42]. Additionally, C1GALT1 has been shown to modify O‐glycosylation of receptor tyrosine kinases, such as MET with the enhancement of HGF‐induced activation in hepatocellular carcinoma [43]. Likewise, our findings suggest C1GALT1 may impact OS progression by influencing key receptors like MET, contributing to poor prognosis and treatment resistance [44, 45]. Our findings provide the first experimental evidence that the presence of C1GALT1‐mediated O‐glycans on ABCC1 affects doxorubicin efflux and sensitivity in OS. This study reveals the novel prognostic significance of C1GALT1 in OS and offers insights into its role, along with ABCC1, in drug resistance. These findings may aid in developing new therapeutic strategies for OS.

## Author contributions statement

N‐YL and M‐CH designed the study. C‐WL and H‐HC collected clinical patients and samples. C‐WL, C‐HC, H‐HC and M‐CL contributed to clinical data analysis. Y‐HT, J‐HH, J‐AL, H‐LC, W‐LC and C‐CC conducted the experiments and analyzed the data. N‐YL and M‐CH prepared the manuscript. N‐YL supervised the study. All authors approved the final version of the article including the authorship list.

## Supporting information


**Figure S1.** ITZ dose‐dependent inhibition of C1GALT1 in OS cells
**Figure S2.** Validation of doxorubicin‐resistant cell line through expression analysis of genes associated with doxorubicin resistance
**Figure S3.** High‐level C1GALT1 expression predicts poor survival in osteosarcoma tissue array (OSCHS801MSur)
**Figure S4.** Silencing efficiency of ABC transporters by shRNA in doxorubicin‐selected cells
**Figure S5.** Confirmation of efficiency of ABCC1 silencing in OS cells
**Figure S6.** Kaplan–Meier survival analysis and C1GALT1 mRNA expression levels in osteosarcoma patients from three public GEO datasets
**Figure S7.** C1GALT1 overexpression enhances ABCC1 membrane localization in G292 cells
**Table S1.** Sequences of sh‐targets and primers used
**Table S2.** Demographic details of patients

## Data Availability

The data that support the findings of this study are available from the corresponding author upon reasonable request.
