# Supplementary material for: C1GALT1 expression predicts poor survival in osteosarcoma and is crucial for ABCC1 transporter‐mediated doxorubicin resistance
Source: J Pathol. 2025 Jan 22;265(3):289–301. doi: 10.1002/path.6384 (PMC11794964; doi:10.1002/path.6384)
Supplement: Supplementary file 1 — Figure S1. ITZ dose‐dependent inhibition of C1GALT1 in OS cells Figure S2. Validation of doxorubicin‐resistant cell line through expression analysis of genes associated with doxorubicin resistance Figure S3. High‐level C1GALT1 expression predicts poor survival in osteosarcoma tissue array (OSCHS801MSur) Figure S4. Silencing efficiency of ABC transporters by shRNA in doxorubicin‐selected cells Figure S5. Confirmation of efficiency of ABCC1 silencing in OS cells Figure S6. Kaplan–Meier survival analysis and C1GALT1 mRNA expression levels in osteosarcoma patients from three public GEO datasets Figure S7. C1GALT1 overexpression enhances ABCC1 membrane localization in G292 cells Table S1. Sequences of sh‐targets and primers used Table S2. Demographic details of patients [file PATH-265-289-s001.docx]

**C1GALT1 expression predicts poor survival in osteosarcoma and is crucial for ABCC1 transporter-mediated doxorubicin resistance**

C-W Liu, J-H Huang, H-H Chang *et al. J Pathol* <https://doi.org/10.1002/path.6384>

**Supplementary Figures S1–S7**

**Supplementary Tables S1 and S2**

**Supplementary Figures S1–S7**

*
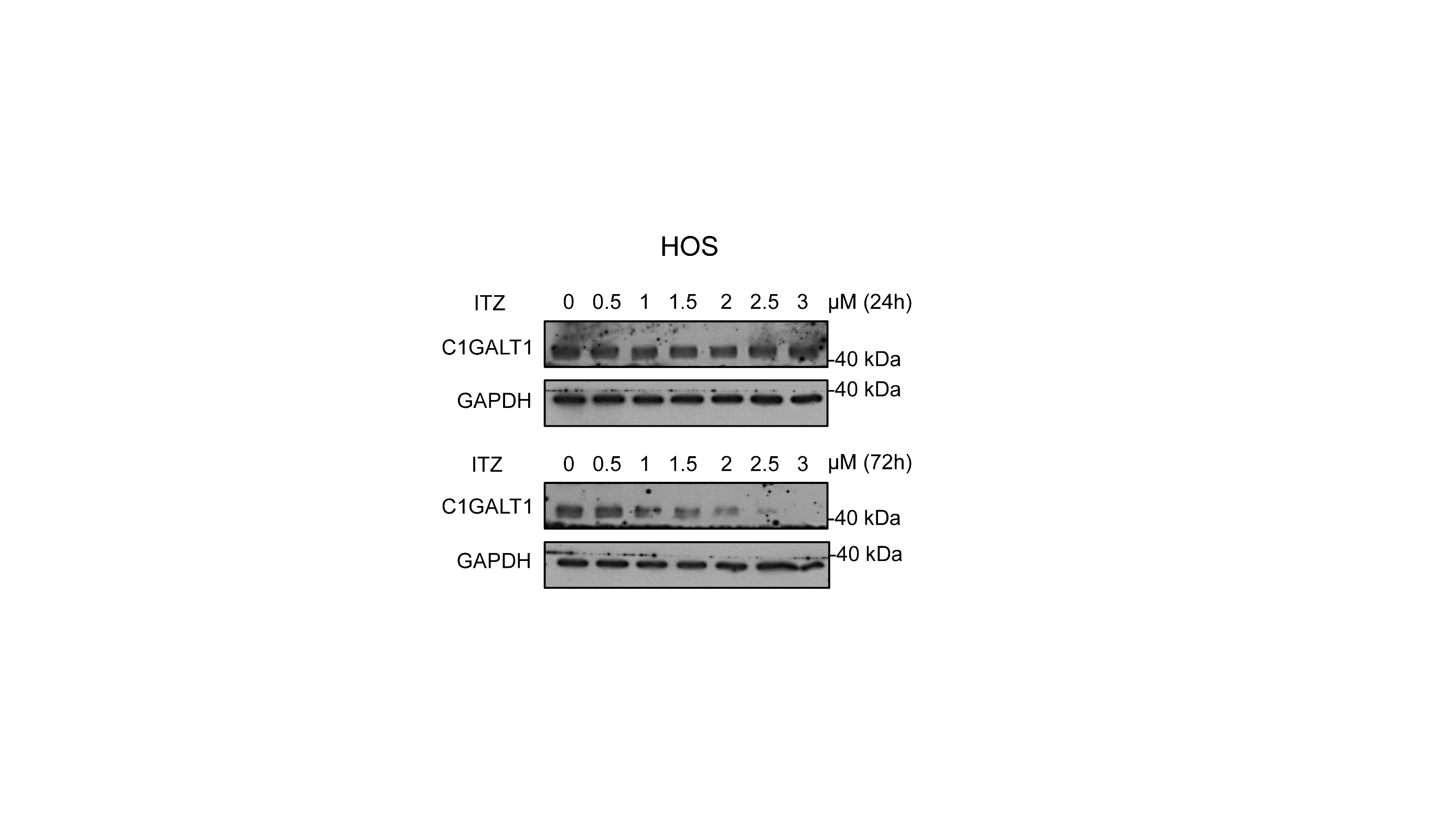
*

**Figure S1. Itraconazole dose-dependent inhibition of C1GALT1 in OS cells.** Western blot analysis of C1GALT1 expression in HOS cells treated with varying concentrations of itraconazole (0, 0.5, 1, 1.5, 2, 2.5, and 3 µM) for 24 and 72 h. The 3-µM dose completely abolished C1GALT1 expression, while 2.5 µM effectively inhibited expression without total suppression. GAPDH was used as loading control.


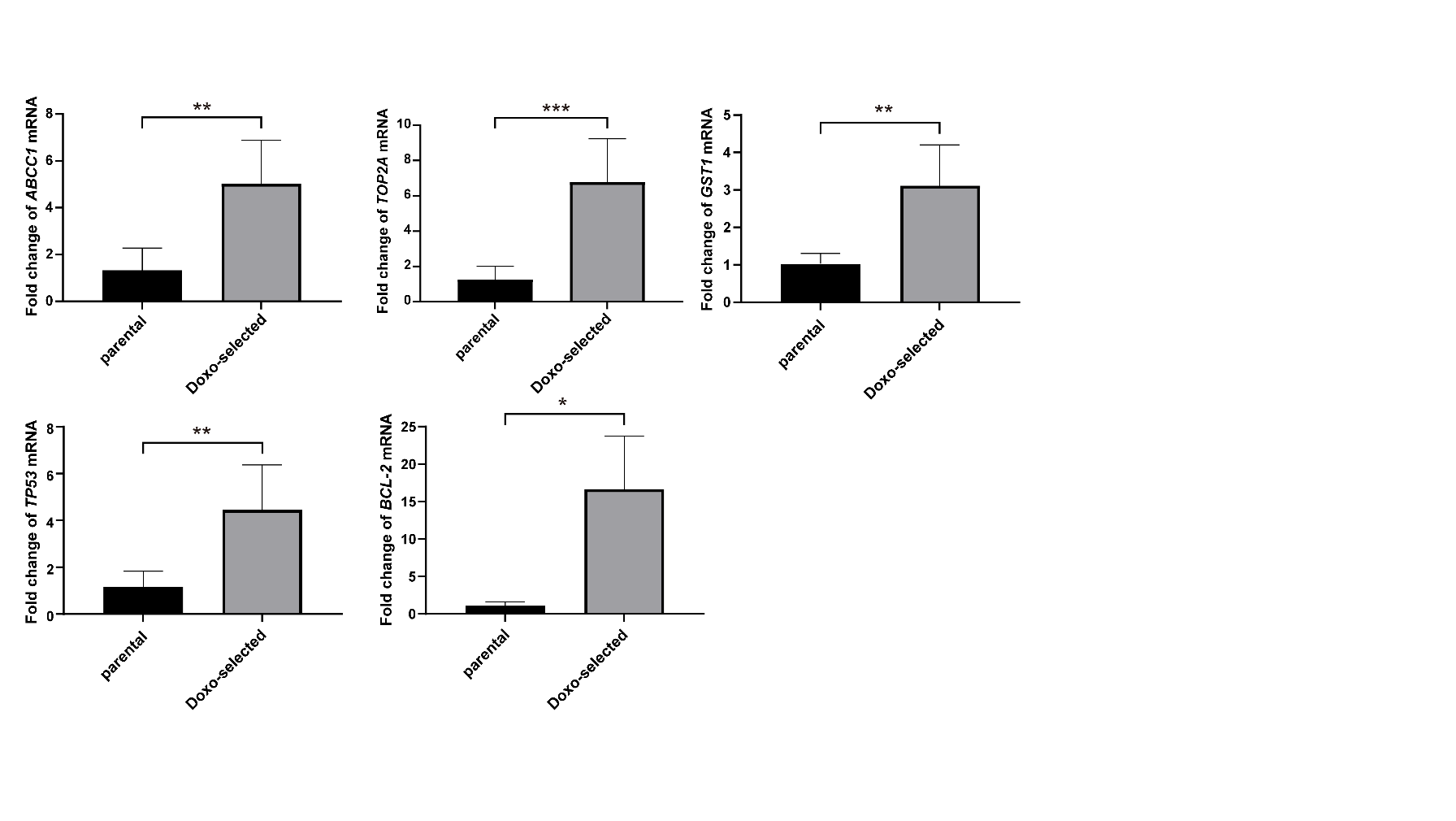


**Figure S2. Validation of doxorubicin-resistant cell line through expression analysis of genes associated with doxorubicin resistance.** Fold-changes in mRNA levels for *ABCC1*, *TOP2A*, *TP53*, *BCL2*, and *GST1* were measured in doxorubicin-selected cells compared to parental cells, showing significant upregulation in resistant cells. Statistical significance is indicated as **p* < 0.05, ***p* < 0.01, ****p* < 0.001.


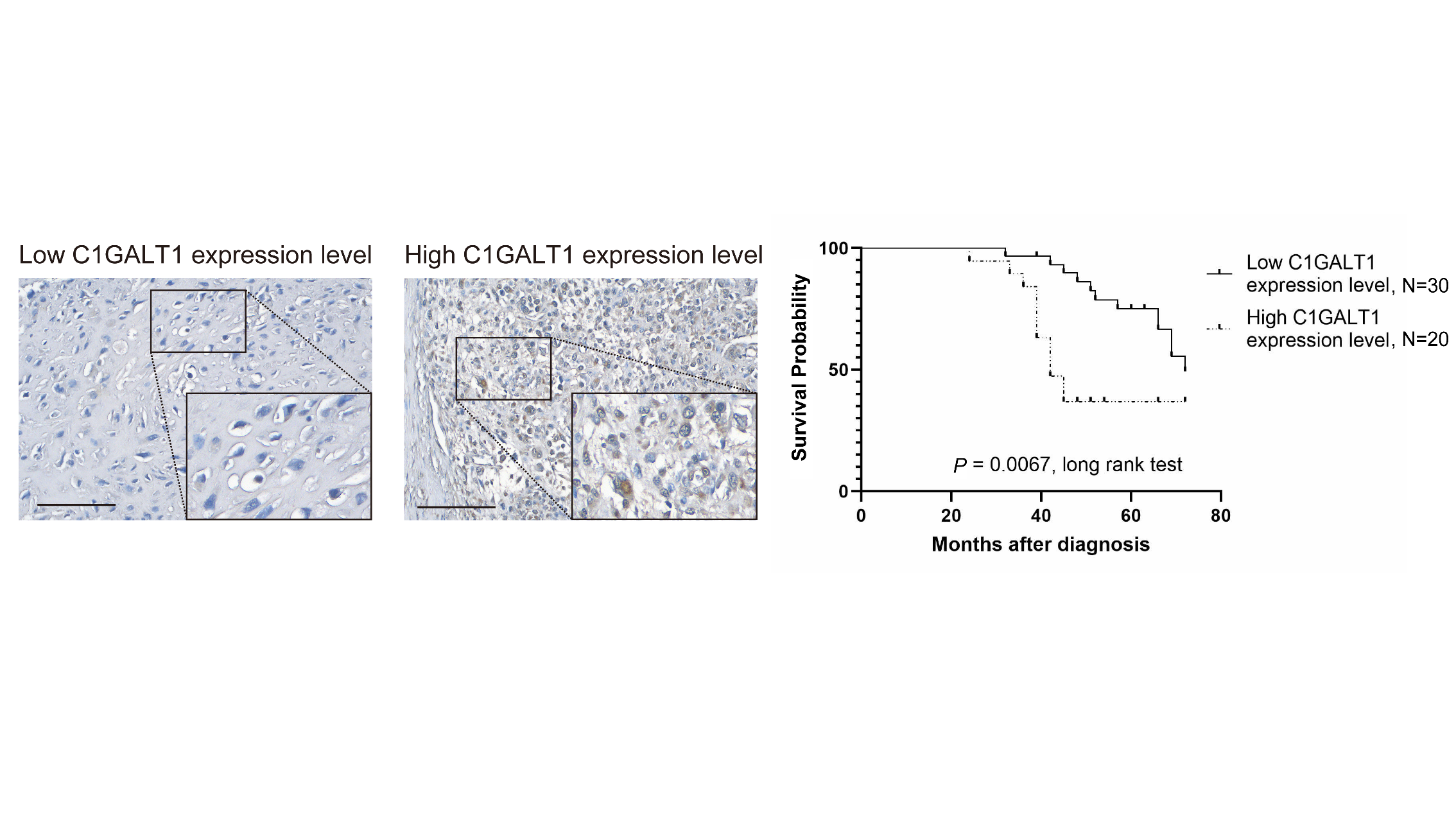

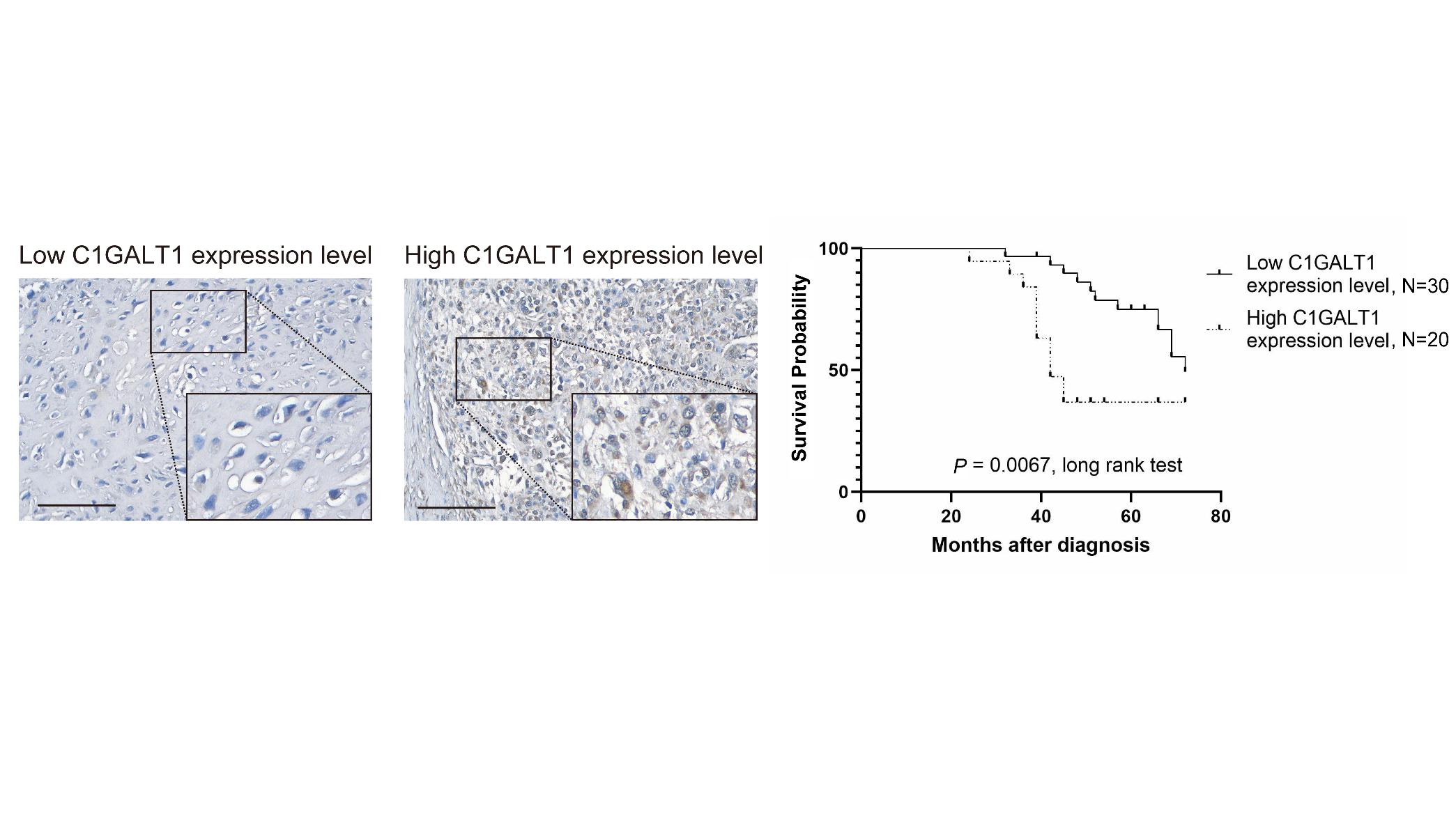


**Figure S3. High-level C1GALT1 expression predicts poor survival in osteosarcoma tissue array (OSCHS801MSur).** Representative immunohistochemistry images showing low and high C1GALT1 expression in osteosarcoma tissue samples, along with Kaplan–Meier survival analysis for patients stratified by C1GALT1 expression levels. This analysis includes a tissue array cohort of 50 osteosarcoma patients (OSCHS801MSur). Patients with high C1GALT1 expression (*N* = 20) demonstrate significantly poorer survival compared to those with low expression (*N* = 30), as shown by the Kaplan–Meier curve (*p* = 0.0067, log-rank test).


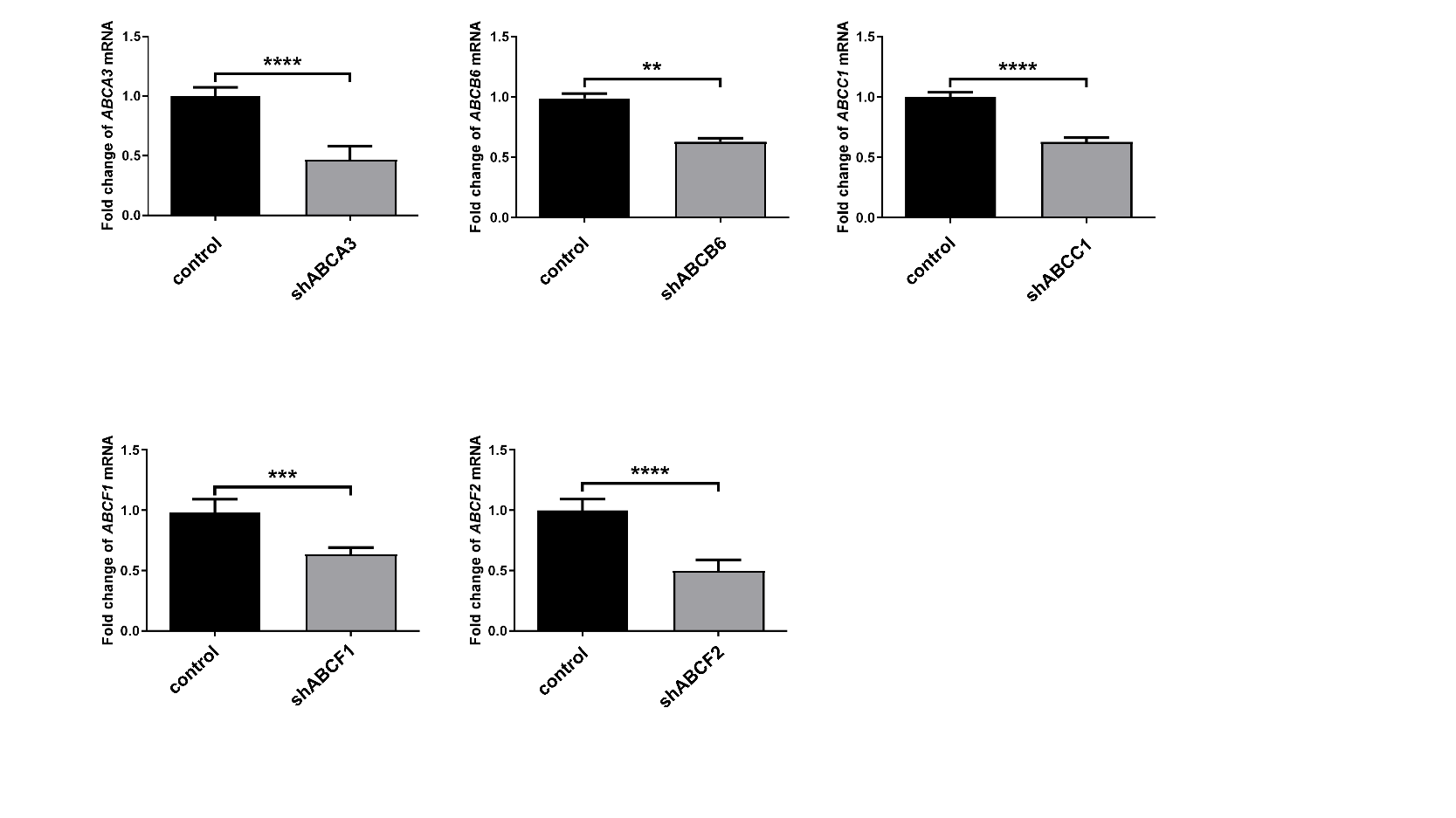


**Figure S4. Silencing efficiency of ABC transporters by shRNA in doxorubicin-selected cells.** RT-qPCR analyses showing mRNA expression levels for ABC transporters involved in doxorubicin selection. Cells transfected with shRNA targeting *ABCA3*, *ABCB6*, *ABCC1*, *ABCF1*, and *ABCF2* displayed a significant reduction in the expression of these genes compared to control cells. Statistical significance is indicated as ***p* < 0.01, ****p* < 0.001, *****p* < 0.0001.


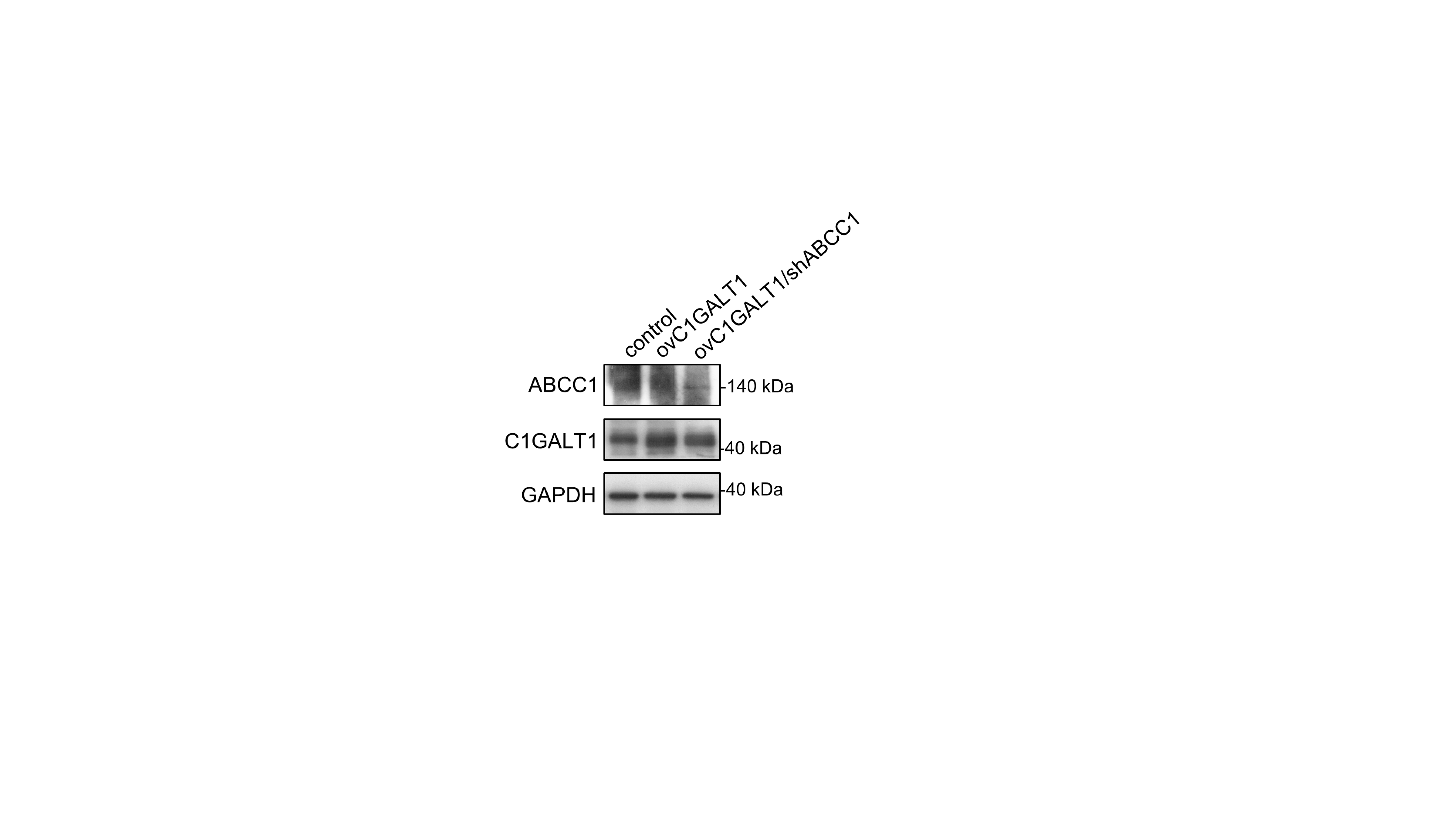


**Figure S5. Confirmation of efficiency of ABCC1 silencing in OS cells.**

Western blot analysis for C1GALT1 and ABCC1 protein levels in OS cells under different conditions: control, *C1GALT1* overexpression (ovC1GALT1), and combined *C1GALT1* overexpression with *ABCC1* silencing (ovC1GALT1/shABCC1). GAPDH served as loading control.


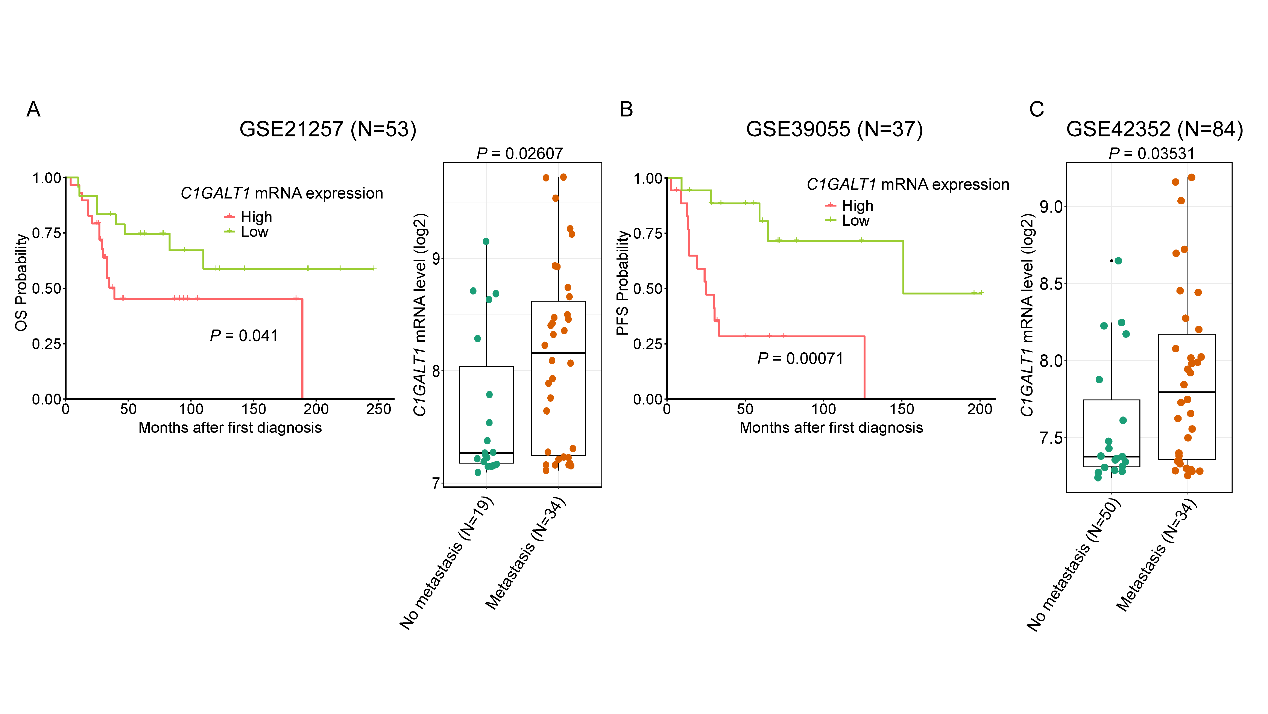


**Figure S6. Kaplan–Meier survival analysis and C1GALT1 mRNA expression levels in osteosarcoma patients from three public GEO datasets.** (A) Overall survival (OS) probability in GSE21257 cohort (*N* = 53), with high C1GALT1 expression correlating with reduced survival (*p* = 0.041). (B) Progression-free survival (PFS) probability in GSE39055 cohort (*N* = 37), showing a significant association between high C1GALT1 expression and shorter PFS (*p* = 0.00071). (C) C1GALT1 expression levels in GSE42352 cohort (*N* = 84), with higher expression observed in patients who developed metastasis within 5 years (*p* = 0.03531). The statistical analyses were performed using R version 4.4.1 software. Survival curves were estimated using the Kaplan–Meier method, and the differences between group curves were compared using the log-rank test using the R packages survival and survminer.


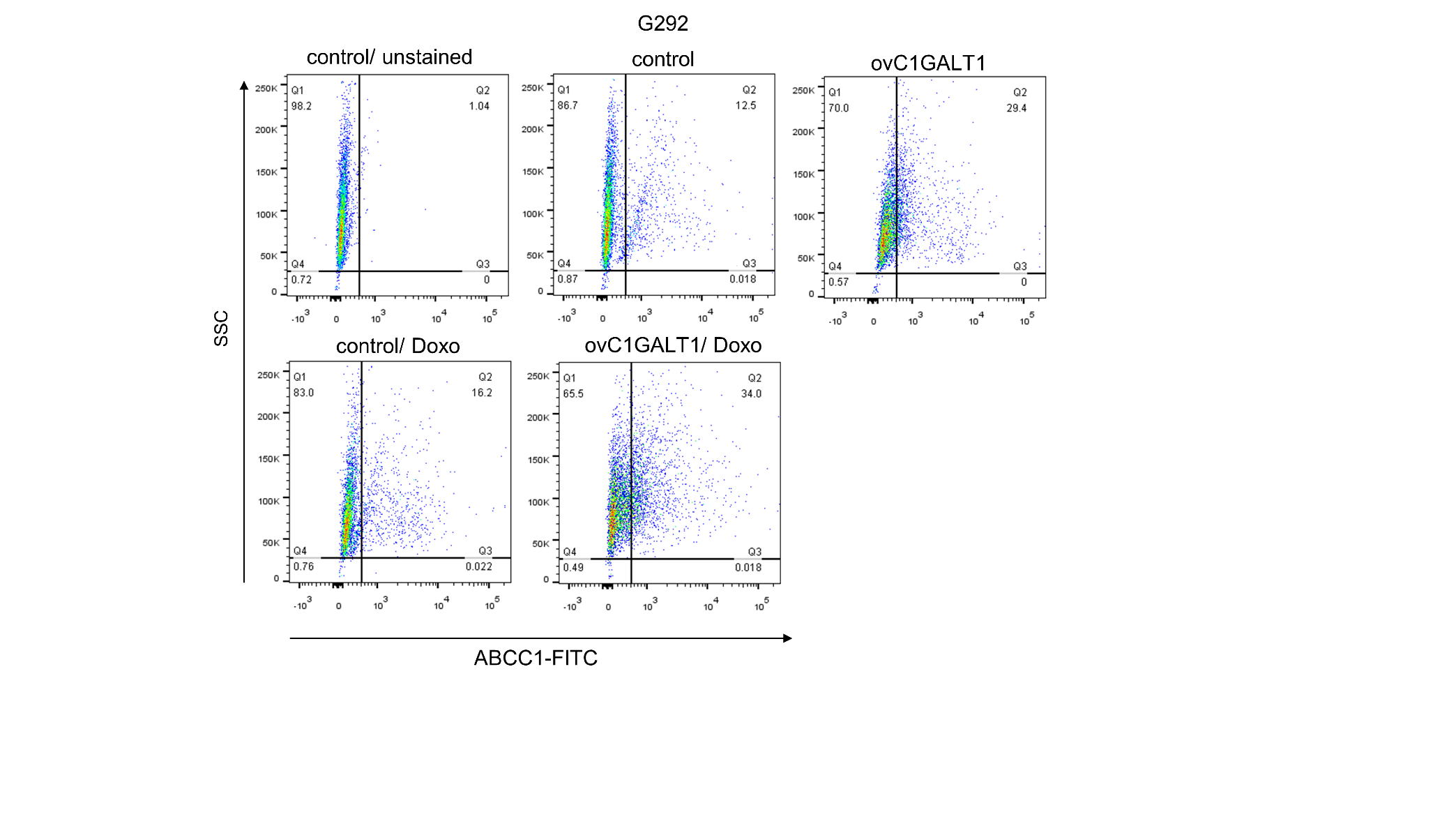


**Figure S7. C1GALT1 overexpression enhances ABCC1 membrane localization in G292 cells.** Flow cytometry analysis showing effect of C1GALT1 overexpression on ABCC1 localization on plasma membrane in G292 cells. Cells were analyzed under control conditions (unstained, untreated, and doxorubicin-treated) and with *C1GALT1* overexpression (ovC1GALT1) in presence or absence of doxorubicin (Doxo).

**Supplementary Tables S1, S2**

**Table S1. Sequences of sh-targets and primers used.**

Target sequences for each candidate are as follows:

shC1GALT1 (5’ CCCAGCCTAATGTTCTTCATA 3’)

shABCA3 (5’ GTTCAGTTACACACGGAGAAA 3’)

shABCB6 (5’ GAACCAAGTTTCGTCGTGCTA 3’)

shABCC1 (5’ CCTCTCAGTGTCTTACTCATT 3’)

shABCF1 (5’ CGGAGAAGAATCGGATCAATA 3’)

shABCF2 (5’ CGTTATGGCCTCATTGGTTTA 3’)

The primer sequences for assessing each mRNA are as follows:

Human *C1GALT1*:

Forward: 5’-TGGGAGAAAAGGTTGACACC-3’

Reverse: 5’-CTTTGACGTGTTTGGCCTTT-3’

Human *GALNT1*:

Forward: 5’-ATGGCCCAGTTACAATGCTC-3’

Reverse: 5’-ATATTTCTGGCAGGGTGACG-3’

Human *GALNT2*:

Forward: 5’-AAGGAGAAGTCGGTGAAGCA-3’

Reverse: 5’-TTGAGCGTGAACTTCCACTG-3’

Human *GCNT1*:

Forward: 5’-TATCTCTGGGCCACCATCCA-3’

Reverse: 5’-GTTCAAGTCACCAGCTCCGA-3’

Human *GCNT3*:

Forward: 5’-GGCAGTGCTTCAGGCTATTC-3’

Reverse: 5’-GGCATACACAGCTCGCAGTA-3’

Human *GCNT4*:

Forward: 5’-AGACGGTGAAACCCCCAAAC-3’

Reverse: 5’- CCCGAATCAAGGTAGCCCAA-3’

Human *ACTB*:

Forward: 5’-CGTGCGTGACATTAAGGAGA-3’

Reverse: 5’-GAAGGAAGGCTGGAAGAGTG-3’

β-actin was utilized as a reference to normalize the loaded cDNA amounts.

**Table S2. Demographic details of patients.**

|  | | | | |  |
| --- | --- | --- | --- | --- | --- |
| Characteristics | All patients  (*N* = 92) | Patients without tumor sample  (*N* = 63) | Patients with tumor sample  (*N* = 29) | *P* value  (with versus without tumor sample) | |
| Age at diagnosis (years) | 13.2 (5.0–18.0) | 13.3 (5.0–18.0) | 13.0 (6.0–17.0) | 0.7479 | |
| Sex |  |  |  | 0.4475 | |
| Male | 56 | 40 (63.5%) | 16 (55.2%) |  | |
| Female | 36 | 23 (36.5%) | 13 (44.8%) |  | |
| Primary location |  |  |  | 0.6069 | |
| Femur | 51 | 33 (55%) | 18 (62.0%) |  | |
| Tibia | 24 | 18 (30%) | 6 (20.7%) |  | |
| Humerus | 6 | 3 (5%) | 3 (10.3%) |  | |
| Others | 11 | 6 (10%) | 2 (7.0%) |  | |
| Location within long bone |  |  |  | 0.8754 | |
| Distal | 54 | 38 (61.3%) | 18 (62.1%) |  | |
| Proximal | 31 | 24 (38.7%) | 10 (34.5%) |  | |
| Staging at diagnosis |  |  |  | 0.1967 | |
| Localized disease | 88 | 40 (93%) | 10 (90.9%) |  | |
| Metastatic disease | 4 | 3 (7%) | 1 (9.1%) |  | |
| Serum ALP at diagnosis | 697.9 | 707.8  (116.0–9550.0) | 681.3  (106.0–2266.0) | 0.9216 | |
| Serum LDH at diagnosis | 541.4 | 558.7  (72.0–3110.0) | 513.7  (173.0–1173.0) | 0.6642 | |
| Histological type |  |  |  | 0.2606 | |
| Osteoblastic | 62 | 41 (71.9%) | 21 (72.4%) |  | |
| Chondroblastic | 7 | 5 (8.8%) | 2 (6.9%) |  | |
| Fibroblastic | 3 | 3 (5.3%) | 0 (0.0%) |  | |
| Others | 20 | 8 (14.0%) | 6 (20.7%) |  | |
| Operation type |  |  |  | 0.0452^*^ | |
| Amputation | 10 | 10 (17.2%) | 0 (0.0%) |  | |
| Limb-sparing surgery | 75 | 47 (81.0%) | 28 (96.6%) |  | |
| Recurrent after therapy |  |  |  | 0.1445 | |
| Yes | 42 | 32 (49.2%) | 19 (65.5%) |  | |
| No | 50 | 31 (50.8%) | 10 (34.5%) |  | |
| Overall survival  (until last follow-up) |  |  |  | 0.1766 | |
| Survived | 54 | 34 (54.0%) | 21 (72.4%) |  | |
| Expired | 37 | 29 (46.0%) | 8 (27.6%) |  | |
